# Detection and Isolation of Sindbis Virus from Field Collected Mosquitoes in Timimoun, Algeria

**DOI:** 10.3390/v14050894

**Published:** 2022-04-25

**Authors:** Nazli Ayhan, Aissam Hachid, Laurence Thirion, Kamel Eddine Benallal, Laura Pezzi, Fayez Ahmed Khardine, Chahrazed Benbetka, Sihem Benbetka, Zoubir Harrat, Remi Charrel

**Affiliations:** 1 Unité des Virus Emergents, UVE: Aix Marseille Université, IRD 190, Inserm 1207, AP-HM Hôpitaux Universitaires de Marseille, 13005 Marseille, France; nazliayhann@gmail.com (N.A.); laurence.thirion@ird.fr (L.T.); laura.pezzi3@studio.unibo.it (L.P.); 2 Laboratoire des Arbovirus et Virus Emergents, Institut Pasteur d’Algérie, Algiers 16096, Algeria; hachidaissam1@gmail.com (A.H.); khardine_78@yahoo.fr (F.A.K.); chahrazed.benbetka@yahoo.fr (C.B.); 3 Faculté de Pharmacie, Université d’Alger1, Algiers 16002, Algeria; 4 Laboratoire d’Eco-épidémiologie Parasitaire et Génétique des Populations, Institut Pasteur d’Algérie, Algiers 16096, Algeria; benallalkamel4@yahoo.fr (K.E.B.); zharrat@gmail.com (Z.H.); 5 Biology Department, Faculty of Sciences, Université Mohamed Bouguerra, Boumerdesse 35000, Algeria; arnsisi@yahoo.fr

**Keywords:** Sindbis virus, alphavirus, *Togaviridae*, *Culex pipiens*, *Culex perexiguus*, arbovirus, Algeria

## Abstract

Sindbis virus (SINV) is a zoonotic alphavirus (family *Togaviridae*, genus *Alphavirus*) that causes human diseases in Africa, Europe, Asia, and Australia. Occasionally, SINV outbreaks were reported in South Africa and northern Europe. Birds are the main amplifying hosts of SINV, while mosquitoes play the role of the primary vector. *Culex* mosquitoes were collected in Algeria and subsequently tested for SINV. SINV RNA was detected in 10 pools out of 40, from a total of 922 mosquitoes tested. A strain of SINV was isolated from a pool displaying high viral load. Whole-genome sequencing and phylogenetic analysis showed that the SINV Algeria isolate was most closely related to a Kenyan strain. This was the first record of SINV in Algeria and more broadly in northwestern Africa, which can be a potential risk for human health in the circulating area. Further studies are needed to measure the impact on public health through seroprevalence studies in Algeria.

## 1. Introduction

Mosquito-borne alphaviruses such as chikungunya, o’nyong-nyong, Mayaro, Ross River, and Sindbis (SINV) viruses can cause large outbreaks of human febrile illness in wide geographical areas. SINV (family *Togaviridae*, genus *Alphavirus*) circulates in Africa, Europe, Asia, and Australia, and SINV human infections were reported in South Africa and northern Europe [1]. It is an enveloped virus with positive single-stranded RNA genome of 11.7 kb coding for four nonstructural (NSP1-4) and five structural (C, E3, E2, 6K, and E1) proteins [2].

SINV was first isolated in 1952 near Cairo, Egypt, during an epidemic of febrile illness with rash and arthritis that was associated with mosquito bites [3]. Since then, SINV was reported in South Africa [4], Europe [5,6], China [7], and Australia [8]. SINV has caused several outbreaks in Finland, Sweden, and South Africa [1] during which patients presented with a rash, fever, and joint inflammation together with fatigue and headache [9]. SINV-related disease in humans was called Pogosta disease in Finland, Ockelbo disease in Sweden, and Karelian fever in Russia [1].

Mosquitoes play a major role as vector species for SINV transmission, while birds are the natural reservoir. Ornithophilic *Culex (Cx)* mosquitoes are the primary enzootic vectors of SINV in different geographical regions [1]. SINV has been isolated/detected from *Culex torrentium/pipiens, Cx. theileri, Cx. perexiguus, Cx. univittatus, Culiseta morsitans*, and a variety of Aedes mosquitoes within the subgenus Ochlerotatus. Strains of SINV have been isolated from mosquitoes in Sweden, Norway, Finland, Germany, Russia, Azerbaijan, South Africa, Australia, Philippines, Israel, Egypt, Saudi Arabia, and Kenya [1,10,11].

Birds, particularly those belonging to *Passeriformes* and *Anseriformes* taxonomic orders, are the amplifying hosts of SINV [1]. It appears that viral spread between continents is largely mediated by bird migrations [12]. SINV-specific antibodies have been found in a large diversity of bird species in UK [13], Finland [14], and Sweden [15].

SINV strains can be discriminated into six genotypes. To date, all human cases have been caused by strains belonging to genotype 1, which circulate in Africa and Europe [1]. Surveillance data from Finland and Sweden suggest an increase of SINV infections in northern Europe [1].

In North Africa, the only evidence related with SINV circulation is a seroprevalence study in birds from Morocco in 1966 [16]. To the best of our knowledge, since then, there are no circulation records of SINV in either patient samples, vector mosquito, or host bird species.

In this study, we describe the first detection and isolation of SINV from *Culex* mosquitoes collected in three different sites of southwestern Algeria in 2017.

## 2. Materials and Methods

### 2.1. Mosquito Sampling

**Figure 1:**
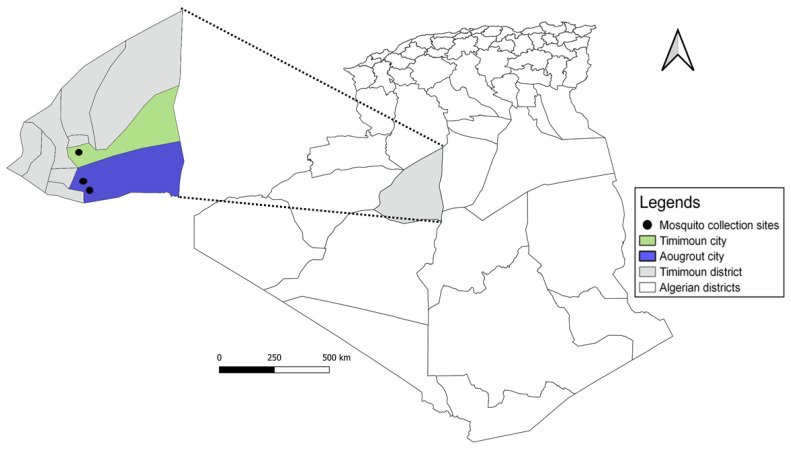
Mosquito collection sites in Algeria.

In September 2017, an entomological survey was carried out in three different regions of the Timimoun district, located 1400 km southwest of Algiers, the capital city. Southwestern Algeria is characterized by a Saharan climate and its well-known oases, many of which are designated as wetlands of international importance [17]. Mosquitoes were captured using five CDC-light traps (1012 model; J. W. Hock Co., Gainesville, FL, USA) near human habitations and in animal shelters (sheep, goats, and chickens) during seven consecutive nights in three sites (Figure 1): (i) Timimoun city (29°14′20.10″ N, 0°12′30.97″ E), an urban oasis site; (ii) Aougrout village (28°41′55.62″ N, 0°20′29.57″ E), a suburban oasis site, and (iii) a sewage discharge (28°43′1.63″ N, 0°18′14.98″ E) (rural site). The two last sites are near each other and are almost 70 km away from the Timimoun city site.

Each day, trapped mosquitoes were pooled by sex, species, date, and site of capture. The morphological identification of the captured mosquitoes to species level was accomplished on a refrigerated tray using identification keys of Schaffner et al. [18]. The number of mosquitoes per pool ranged from 2 to 45. All specimens were stored in liquid nitrogen until processed.

### 2.2. SINV Screening

Mosquito pools were homogenized as previously described [19], and viral RNA was extracted by using a QIAamp Viral RNA Mini Kit (Qiagen, Hilden, Germany) according to the manufacturer’s instructions. Only females were tested for SINV RNA detection.

In-house SINV-specific real-time RT-qPCR assay was used to screen mosquito pools. To design specific primers and probes, SINV sequences were downloaded from GenBank and aligned using ClusterW in MEGA6 software. Accordingly, a forward primer (5′-CCA AGA GCC TGC CCC TRT TC-3′), a reverse primer (5′-CAG GCG GGC CAT CTT CT-3′), and a probe (5′-FAM-ACC GCC AAG GCT A-MGB-3′) were designed manually, targeting a conserved region of nonstructural polyprotein gene.

PCR amplification was performed using a SuperScript III Platinum One-Step qRT-PCR (ThermoFisher Scientific, Waltham, MA, USA) kit with 20 µL reaction mix and 5 µL of RNA. Total volume was subjected to real-time RT-qPCR amplification using the following cycling conditions: 50 °C for 15 min, 95 °C for 2 min, 45 cycle for 95 °C for 15 s, and 58 °C for 45 min on an Applied Biosystems 7500 real-time PCR thermocycler (ThermoFisher Scientific, Waltham, MA, USA).

### 2.3. Virus Isolation

The pool exhibiting the highest viral load (lowest cycle threshold (Ct) value) was selected for attempting virus isolation in cell culture. A volume of 200 µL of mosquitoes’ homogenate was inoculated on Vero African green monkey kidney cells (Vero (ATCC CCL81)). The T-25 cell culture flask was incubated at room temperature for one hour; then, cells were washed with Hank’s Balanced Salt Solution and 5 mL of fresh maintenance medium (MEM supplemented with 2% FBS, 1% penicillin–streptomycin, 1% (200 mM) L-glutamine, 1% Kanamycin, and 3% Amphotericin B) was added. Cells were incubated at 37 °C in a 5% CO_2_ atmosphere and examined daily for the presence of the cytopathic effect (CPE) and passaged successively three times. After each passage, 200 μL of supernatant medium was collected from the culture and analyzed using the SINV specific RT-qPCR assay.

### 2.4. Sequencing

Passage 1, corresponding to the inoculation of the pool P29/17, was selected for complete genome sequencing using Next-Generation Sequencing (NGS). A total of 200 μL of supernatant was incubated at 37 °C for 7 h with 25 U of Benzonase (Novagen, Houston, TX, USA) and MgCl_2_. RNA extraction was performed using a Viral RNA minikit (Qiagen, Hilden, Germany) on the BioRobot EZ1-XL Advanced (Qiagen, Hilden, Germany). Random two-step RT-PCR was realized using tagged random primers. A ProtoScript^®^ II Reverse Transcriptase kit (New England Biolabs, Ipswich, MA, USA) was used for reverse transcription with specific primers and Platinum^®^ Taq High Fidelity polymerase enzyme (Thermo Fisher Scientific, Waltham, MA, USA) for amplification [20]. After quantification using a Qubit^®^ dsDNA HS Assay Kit and a Qubit 2.0 fluorometer (ThermoFisher Scientific, Waltham, MA, USA), amplicons were split (sonication) into 200 bp-long fragments. Libraries were built by adding barcodes for sample identification and primers to fragmented DNA using the AB Library Builder System (ThermoFisher Scientific, Waltham, MA, USA). To pool equimolarly the barcoded samples, a quantification step by real-time PCR using an Ion Library TaqMan™ Quantitation Kit (Thermo Fisher Scientific, Waltham, MA, USA) was realized. An emulsion PCR of the pools and loading on 520 chip was realized using the automated Ion Chef instrument (ThermoFisher, Waltham, MA, USA). Sequencing was performed using S5 Ion torrent technology (Thermo Fisher Scientific) following the manufacturer’s instructions. Reads were trimmed (reads with quality score <0.99 and length <100 bp were removed, and the first and last 30 nucleotides were removed from the reads) and de novo contigs were produced by CLC genomics workbench software v.21 (Qiagen, Hilden, Germany). These contigs were aligned to determine the best reference sequence(s). CLC genomics workbench was used to process reads. Reads longer than 30nts were trimmed using the CLC genomics workbench with 99% quality per base and mapped to a reference sequence (GenBank acc no. KY616987). Reads mapped to the reference sequence for at least 50% of its length with a minimum of 80% identity to the reference were used.

### 2.5. Genetic and Phylogenetic Analysis

SINV-Algeria_2017 sequence was aligned using ClustalW together with other selected SINV sequences retrieved from GenBank. The nucleic acid alignment was translated into amino acid sequences, verified and refined manually using MEGA 6 software [21]. Based on partial nucleotide sequence of the *E2* gene, the most variable alphavirus gene encoding one of the envelope proteins, a maximum likelihood tree was computed using the Kimura 2-parameter model, the nearest neighbor interchange tree search method, and 1000 bootstrap replicates.

## 3. Results

### 3.1. Mosquito Trapping and SINV Screening

A total of 1145 mosquitoes (922 females, 223 males) were collected in the three regions of Timimoun district, in southwest of Algeria (Figure 1).

They consisted of 1138 *Culex* mosquitoes of which 598 were identified as *Cx. perexiguus* (489 females, 109 males) and 540 as *Cx. pipiens* (427 females, 113 males); the remaining were six *Anopheles d’thali* (five females, one male) and one *Anopheles rhodesiensis rupicolus* (Table 1).

After morphological identification of mosquito species, 40 pools were prepared (Table 2); 10 were found to contain SINV RNA with Ct values ranging from 15 to 38 (Table 2). All the SINV RNA-positive pools had been collected in Aougrout except for one collected in Timimoun city. Six positive pools consisted of *Cx. Pipiens*, and four pools contained *Cx. perexiguus* (Table 2).

### 3.2. Virus Isolation

Pool 29/17 showed a clear cytopathic effect (CPE) at day 3 post-infection at passage 1. Three passages were done for large production of the virus to constitute a batch of 80 vials for the European Virus Archive-GLOBAL (*EVAg*, https://www.european-virus-archive.com/) [22] collection according to the quality management system requirements. This material will be available for academia and industry under the EVA policy and canbe found in the online catalog under reference 001V-04477.

### 3.3. Sequencing and Phylogenetic Analysis

**Figure 2:**
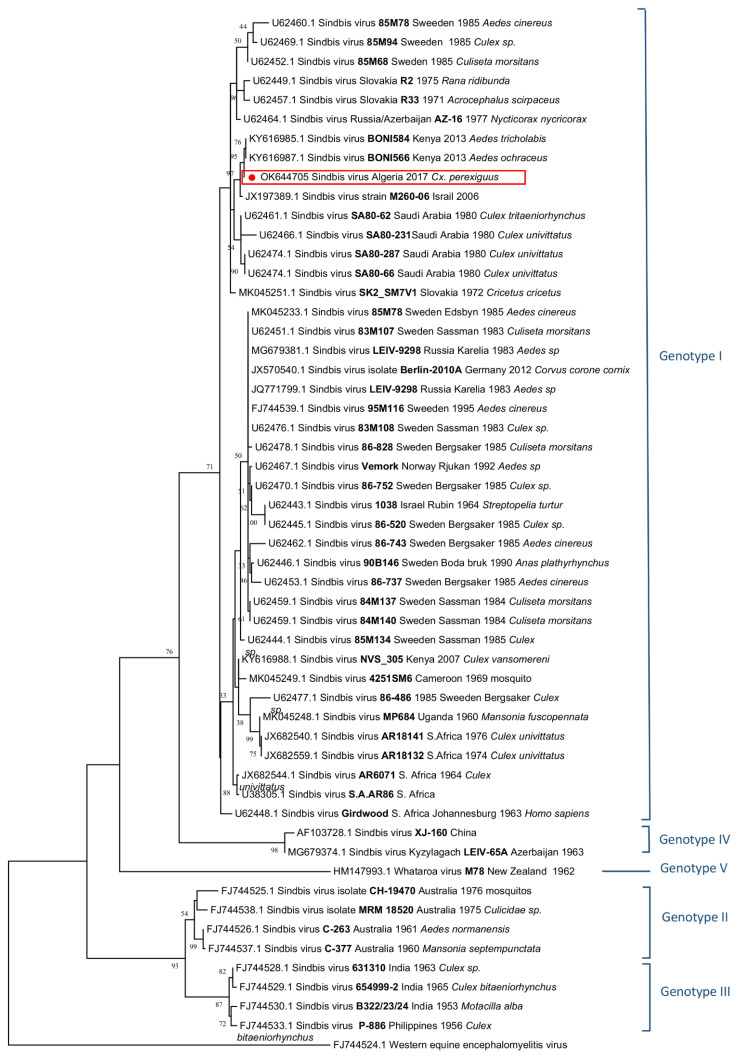
Phylogenetic analysis showing the genetic relationships of SINV genotypes based on partial nucleotide sequencing of the E2 gene.

SINV genome (SINV-Algeria_2017-*Cx. perexiguus*) of the pool 29/17 consisted of 11,522 nucleotides with two open reading frames (GenBank acc no. OK644705). Complete genome analysis with BLAST showed that SINV-Algeria_2017-*Cx. perexiguus* strain belonged to genotype 1 and was most closely related (98.53% nucleotide identity) to the SINV Kenya Boni forest strain (GenBank acc no. KY616987) identified in Kenya in 2013 [10]. Phylogenetic analysis performed using the E2 gene sequence provided topologies that were similar to that observed with complete coding sequence. SINV-Algeria_2017-*Cx. perexiguus* belonged to genotype 1 that contains strains from Africa and Europe (Figure 2).

## 4. Discussion

Here, we report the detection, isolation, and genetic characterization of SINV from mosquitoes collected in Algeria. One strain named SINV-Algeria_2017-*Cx. perexiguus* was isolated from a pool containing 49 *Cx. perexiguus* mosquitoes trapped in the Aougrout oasis, Timimoun district. Previously in this region, the West Nile virus has been detected in *Cx. perexiguus* [23], and WNV human cases were reported [24,25].

To our knowledge, this is the first direct evidence of the presence of SINV in Algeria. In the Maghreb region, only one study conducted in 1966 in Morocco reported that out of 91 birds from different species, four barn swallows (*Hirundo rustica*), which migrate from Europe to Africa through North Africa in late summer and autumn, were found positive for the presence of SINV antibodies [16].

Birds of the orders Passeriformes, Galliformes, and Anseriformes exhibit high viremia after experimental infection with SINV, specifically in young individuals [26]. In Algeria, many passerine species have been spotted in Timimoun areas [27]. Ornithophilic mosquitoes such as *Cx. pipiens* and *Cx. torrentium* are the vectors for transmitting the virus to susceptible hosts. In addition, the ability of *Cx. pipiens* collected in the Timimoun region to transmit efficiently West Nile and Rift valley fever viruses has been experimentally proven [28].

In our study, SINV RNA was detected in pools consisting of *Cx. perexiguus* and *Cx. pipiens* that had been already identified as the vector of SINV in Israel and Egypt. SINV was detected in *Cx. pipiens* in Israel, Saudi Arabia, and Sweden [1,29]. Both species are mainly ornithophilic, although they feed also on humans, allowing the spillover of the virus from bird to human [30].

As expected, SINV-Algeria-2017-*Cx. perexiguus* belonged to genotype 1 that includes strains of Europe (Sweden, Finland, Germany, Norway, Russia) and Africa (Cameroon, Uganda, South Africa) that have been isolated/detected in mosquitoes, birds, and small terrestrial mammals. Genotype 1 also contains human strains accounting for outbreaks in South Africa and Northern Europe [1].

## 5. Conclusions

In conclusion, we have (i) identified SINV in mosquitoes in Algeria. (ii) provided genetic evidence supporting that SINV-Algeria-2017-*Cx. perexiguus* strain belongs to genotype 1, and (iii) identified *Cx. perexiguus* and *Cx. pipiens* as potential vector species of SINV in Algeria. Whether they are the primary vector of SINV should be investigated. Further studies are needed to measure the impact on public health through seroprevalence studies and inclusion of SINV as differential diagnosis of arboviral diseases.

## Figures and Tables

**Table 1:** Mosquito species identification by site of collection.

Species	Site	Females	Males
*Culex perexiguus*	Aougrout (suburban)	461	101
	Timimoun city (urban)	2	0
	Sewage discharge (rural)	26	08
*Culex pipiens*	Aougrout (suburban)	290	65
	Timimoun city (urban)	71	23
	Sewage discharge (rural)	66	25
*Anopheles d’thali*	Aougrout (suburban)	5	1
*Anopheles rhodesiensis rupicolus*		1	0
Total		922	223

**Table 2:** Description of the 10 pools containing SINV RNA.

Pool #	Number of Mosquito/Pool	Collection Site	Morphological Identification	qRT-PCR Sindbis (Ct)
P4/17	30	Augrout	*Cx. perexiguus*	36.9
P5/17	30	Augrout	*Cx. perexiguus*	34.9
P9/17	30	Augrout	*Cx. perexiguus*	27.2
P14/17	30	Augrout	*Cx. pipiens*	38.0
P15/17	30	Augrout	*Cx. pipiens*	36.3
P19/17	30	Augrout	*Cx. pipiens*	37.0
P20/17	30	Augrout	*Cx. pipiens*	35.0
P28/17	30	Augrout	*Cx. pipiens*	28.8
P29/17	49	Augrout	*Cx. perexiguus*	15.0
P32/17	16	Timimoun	*Cx. pipiens*	33.0

## Data Availability

SINV Algeria sequence data submitted to GenBank (acc no. OK644705).
